# The Inducible Accumulation of Cell Wall-Bound *p*-Hydroxybenzoates Is Involved in the Regulation of Gravitropic Response of Poplar

**DOI:** 10.3389/fpls.2021.755576

**Published:** 2021-12-14

**Authors:** Yunjun Zhao, Xiao-Hong Yu, Chang-Jun Liu

**Affiliations:** Brookhaven National Laboratory, Biology Department, Upton, NY, United States

**Keywords:** lignin, *p*-hydroxybenzoate, monolignol *p*-hydroxybenzoyltransferase, tension wood, gravistimulation, hybrid aspen

## Abstract

Lignin in *Populus* species is acylated with *p*-hydroxybenzoate. Monolignol *p*-hydroxybenzoyltransferase 1 (PHBMT1) mediates *p*-hydroxybenzoylation of sinapyl alcohol, eventually leading to the modification of syringyl lignin subunits. Angiosperm trees upon gravistimulation undergo the re-orientation of their growth along with the production of specialized secondary xylem, i.e., tension wood (TW), that generates tensile force to pull the inclined stem or leaning branch upward. Sporadic evidence suggests that angiosperm TW contains relatively a high percentage of syringyl lignin and lignin-bound *p*-hydroxybenzoate. However, whether such lignin modification plays a role in gravitropic response remains unclear. By imposing mechanical bending and/or gravitropic stimuli to the hybrid aspens in the wild type (WT), lignin *p*-hydroxybenzoate deficient, and *p*-hydroxybenzoate overproduction plants, we examined the responses of plants to gravitropic/mechanical stress and their cell wall composition changes. We revealed that mechanical bending or gravitropic stimulation not only induced the overproduction of crystalline cellulose fibers and increased the relative abundance of syringyl lignin, but also significantly induced the expression of *PHBMT1* and the increased accumulation of *p*-hydroxybenzoates in TW. Furthermore, we found that although disturbing lignin-bound *p*-hydroxybenzoate accumulation in the *PHBMT1* knockout and overexpression (OE) poplars did not affect the major chemical composition shifts of the cell walls in their TW as occurred in the WT plants, depletion of *p*-hydroxybenzoates intensified the gravitropic curving of the plantlets in response to gravistimulation, evident with the enhanced stem secant bending angle. By contrast, hyperaccumulation of *p*-hydroxybenzoates mitigated gravitropic response. These data suggest that PHBMT1-mediated lignin modification is involved in the regulation of poplar gravitropic response and, likely by compromising gravitropism and/or enhancing autotropism, negatively coordinates the action of TW cellulose fibers to control the poplar wood deformation and plant growth.

## Significance

Lignin in many plant species is decorated with *p*-hydroxybenzoate. Despite the long history of recognition of its existence in plant cell walls, our understanding of the biological significance of such lignin modification remains largely elusive. In the present study, we explore the potential roles of lignin-bound *p*-hydroxybenzoates in the regulation of gravitropic response with respect to gravistimulation. We revealed that mechanical bending or gravistimulation significantly induced the expression of *p*-hydroxybenzoyl CoA: monolignol *p*-hydroxybenzoyltransferase 1 gene and the consequent accumulation of *p*-hydroxybenzoates in tension wood (TW). Paradoxically, hyperaccumulation of *p*-hydroxybenzoates considerably mitigated gravitropic response of poplar plantlets; conversely, depletion of lignin *p*-hydroxybenzoates enhanced poplar gravitropic response. Our study reveals an adaptive mechanism of poplars in response to the mechanical stress/gravistimulation, in which the induced production of crystalline cellulose fibers and the enhanced accumulation of lignin *p*-hydroxybenzoates might coordinately regulate the gravitropic response process thus controlling the poplar wood deformation and tree architecture. This finding contributes to our understanding of the biological functions of prevalent lignin acylation.

## Introduction

As a sessile organism, the terrestrial plants evolve remarkable abilities in modulating their normal growth to cope with different environmental stresses. In response to gravity or mechanical stress enforced by environmental factors, such as wind or snow, plants slowly re-orient their growth to ensure that most shoots grow up and most roots grow down. This process is termed as gravitropism (Stankovic et al., 1998a,b; Moulia and Fournier, 2009; Bastien et al., 2013). Re-orientation of organ results in a net gravitropic curvature. After the organ curves up, a phase of autotropic straightening or decurving starts at the tip and propagates downward, so that the curvature finally becomes concentrated at the base of the growth zone and steady (Stankovic et al., 1998b; Coutand et al., 2007; Bastien et al., 2013). This decurving process, i.e., the tendency of plants to recover straightness in the absence of any external stimulus is described as autotropism. The gravistimulation-triggered general curving, followed by basipetal straightening to prevent overshoot compose the biphasic process of plant gravitropic response (Stankovic et al., 1998b; Coutand et al., 2007; Bastien et al., 2013). The final set-point angle of an organ is achieved by a composite response of two tropisms, i.e., gravitropism and autotropism.

The molecular basis for the gravitropic movement of angiosperm trees is the formation of specialized secondary xylem, i.e., tension wood (TW) in their branches and/or displaced stems of porous wood with respect to gravity. In the inclined tree, TW is formed on the upper part of the stem, whereas wood formed on the lower side is termed as opposite wood (OW). TW induces tensile force for generating tensile growth stress to pull the inclined stem or lean branches upward, thereby preventing the stem/branch from bending and cracking (Yamamoto, 1998; Yoshida et al., 2002).

A major anatomical characteristic of TW in most angiosperms is the fibers with an additional gelatinous layer (G-layer) at the inner face of the secondary wall (Ghislain and Clair, 2017). The G-layer is composed mostly of highly crystalline cellulose microfibrils that are oriented parallel or nearly parallel to the fiber axis (Timell, 1969; Groover, 2016). The formation of G-layer in TW fibers results in a corresponding decrease in lignin, xylan, and glucomannan (Timell, 1969). In addition, TW lignin is known to have an increased syringyl/guaiacyl (S/G) ratio (Yoshida et al., 2002; Pilate et al., 2004; Ghislain and Clair, 2017). The previous studies revealed that increasing tensile growth stress is correlated with the decrease of cellulose microfibril angle and lignin content, and the increase of α-cellulose content and cellulose crystallinity (Okuyama et al., 1990, 1994). TW fiber walls are diverse in their organization of cell wall layers and lignification. In general, they are classified into three anatomical groups: with lignified G-layer, with non-lignified G-layer, and without G-layer (Ghislain and Clair, 2017). Interestingly, a recent study revealed that lignin in the G-layer does not effectively affect the mechanical action of TW, since the maturation strain of TW is similar between the species with lignified and non-lignified G-layers (Ghislain et al., 2019).

Lignin is a heterogenous aromatic polymer, derived primarily from the oxidative radical coupling of three phenylpropanoid units, *p*-coumaryl alcohol, coniferyl alcohol, and sinapyl alcohol (i.e., monolignols). Three monolignols, after depositing into cell walls, oxidatively couple to each other or to the growing lignin polymer, yielding *p*-hydroxyphenyl (H), guaiacyl (G), and syringyl (S) lignin subunits (Vanholme et al., 2010). Compelling chemical and genetic evidence have revealed that cell wall lignification exhibits considerable plasticity, allowing plants not only to dramatically alter the levels of traditionally abundant subunits but also incorporate “non-traditional” subunits in lignin polymer (Vanholme et al., 2012; Mottiar et al., 2016). Consequently, the lignin structure and composition vary greatly depending on the taxonomic groups, plant species, plant organs, tissues, cell-types, cell wall layers, the stages of plant development, and environmental conditions (Boerjan et al., 2003; Ralph, 2007).

Beyond conventional monolignols, various monolignol conjugates are known to participate in lignification (Ralph, 2010; Vanholme et al., 2012; Mottiar et al., 2016; del Rio et al., 2020). In particular, the naturally occured acylated lignin that has been encountered in various plant species of a wide range of taxa contains the ester-bond linked different carboxylic acid groups. For instance, lignin in kenaf bast fiber and various other plants, such as the monocot palms, abaca (*Musa textilis*), and sisal (*Agave sisalana*) are extensively acetylated (Lu and Ralph, 2002; del Rio et al., 2004, Del Rio et al., 2007; del Rio et al., 2008). Commelinid grass lignin commonly contains *p*-coumarate (*p*CA) pendants that exclusively occur on the γ-hydroxyl of the sidechain of syringyl units (Lu and Ralph, 1999; del Rio et al., 2008, 2012; Karlen et al., 2018).

Analogous to the frequently reported *p*CA ester, *p*-hydroxybenzoate (*p*BA) is found to attach to the lignin *via* ester bond in hardwoods of the *Salicaceae* family, such as willows (*Salix* spp.), poplars and aspens (*Populus* spp.) (Smith, 1955; Landucci et al., 1992; Lu et al., 2004; Morreel et al., 2004a), and the family of *Araliaceae* Aralia cordata (Hibino et al., 1994), as well as in the monocot palms of the large *Arecaceae* family (Lu et al., 2015; Karlen et al., 2018). Moreover, *p*BA also acylates lignin at the γ-hydroxyl of the sidechain of syringyl unit as pendant group and the modification arises through the incorporation of acylated monolignol conjugates (Nakamura and Higuchi, 1978; Lu et al., 2004, 2015; Morreel et al., 2004b). Interestingly, a recent study revealed that the *p*-hydroxybenzoylated lignin was also detected in seagrass *Posidonia oceanica*; and the acylation occurred on both the G- and S-lignin subunits, which is contrary to what is observed in poplar, willow, and palm, where *p*BAs overwhelmingly appear on the S-subunits (Rencoret et al., 2020).

Despite the recognition of the prevalent existence of *p*BAs in the lignin of many plant species, our understanding of the biological significance of lignin-bound *p*BAs remains fragmentary. Quantifying *p*BA levels in hybrid poplar TW reveals that *p*BAs are increased compared with those in OW or normal wood (NW) (Hedenstrom et al., 2009; Foston et al., 2011; Goacher et al., 2021), implicating the potential involvement of the lignin-bound *p*BAs in mechanical stress response or TW formation. Through the functional genomics study, we recently have determined a BAHD family acyltransferase, namely, p-hydroxybenzoyl CoA: monolignol p-hydroxybenzoyltransferase 1 (PtrPHBMT1), from *P. trichocarpa* (Zhao et al., 2021). The characterized enzyme exhibits preferential catalytic activity in conjugating *p*-hydroxybenzoate from its CoA thioester to monolignol sinapyl alcohol. Disruption of *PHBMT1 via* CRISPR-Cas9 gene editing in hybrid poplar nearly completely depleted *p*BA accumulation in the stem lignin; conversely, overexpression (OE) of the gene increased lignin *p*BA accumulation. Furthermore, *in vitro* analysis revealed that elimination of the lignin-bound *p*BAs significantly enhanced the solvent dissolution rate of lignin polymer, suggesting that *p*-hydroxybenzoylated lignin somehow alters its physicochemical properties. As a follow-up, in the present study, we explore the biological functions of PHBMT1-mediated monolignol *p*-hydroxybenzoylation (ultimately the accumulation of lignin *p*BAs) in gravitropic stress response of poplar. We discover that gravistimulation and mechanical bending strongly induce the expression of *PHBMT1* and the accumulation of lignin-bound *p*BAs in TW of poplar; whereas, paradoxically and interestingly, eliminating *PHBMT1* and depleting lignin-bound *p*BAs appear to substantially enhance the stem curving of the plantlets upon gravistimulation; while the OE of *PHBMT1* and hyperaccumulation of *p*BAs mitigate gravitropic response, evident with small stem secant bending angle. We discuss the potential role of PHBMT1-mediated *p*-hydroxybenzoylation in the regulation of gravitropism and autotropism of poplar.

## Materials and Methods

### Plant Materials

Hybrid aspen clone *Populus tremula* x *P. alba* INRA 717-IB4 was used in the study. Two representative CRISPR-Cas9 lines (g1-8 and g1-9), two representative OE lines (OE1 and OE2) that harbor *PtrPHBMT1*, driven by PvPAL2 promoter, were from our previous study (Zhao et al., 2021). The previously characterized transgenic lines and their corresponding wild type (WT) controls that were maintained in the magenta boxes with tissue culture propagation medium without antibiotic selection (2.15 g L^–1^ MS basal salts, 0.25 g L^–1^ MES, 0.1 g L^–1^ myo-inositol, 0.2 g L^–1^ L-glutamine, 0.001 g L^–1^ nicotinic acid, 0.001 g L^–1^ pyridoxine HCl, 0.001 g L^–1^ calcium pantothenate, 0.001 g L^–1^ thiamine HCl, 0.001 g L^–1^ *L*-cysteine, 0.01 g L^–1^ biotin, 0.0005 g L^–1^ IBA, 20 g L^–1^ sucrose and 7 g L^–1^ agar, pH 5.8) were propagated in the same medium by using apical shoot explants to obtain enough biological replicates. After propagation, the poplar plantlets were transferred to soil (PRO-MiX BX, Premier Tech Horticulture, Canada) in pots of 5.5 cm diameter and covered with transparent plastic cups for 1 week to keep the moisture. Then, the plants were uncovered and grown without bundling with supporting poles at 28°C during daytime and 23°C during the night with a long-day regime (14 h light/10 h dark). During the growth period of the plantlets in the experiment, no fertilizers were applied.

### Mechanical Stress, Gravitropic Stimulation, and Tension Wood Induction

One-year-old hybrid aspens in the greenhouse were enforced with mechanical bending for TW induction. The trees were bent with a rope for 10 days. The trees without bending that grew side by side with the treated trees served as the control. After treatment, the trees were harvested and their bark was removed from the curved region, and the differentiating xylem was harvested by light scraping with a razor blade at the upper (TW) and lower (OW) sides of the stem. For trees as the control, the differentiating xylem was harvested in the same internodes as the bent trees. A portion of collected differentiating xylem materials was used for total RNA isolation and real-time quantitative PCR (qRT-PCR) analysis. The rest were used for cell wall preparation and wall-bound phenolic analysis.

**FIGURE 1:**
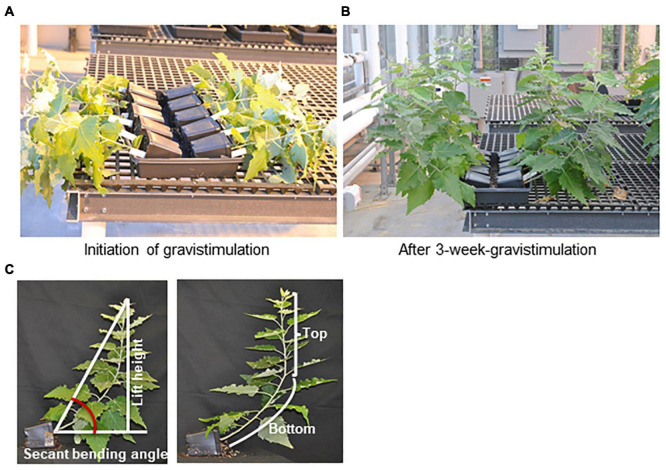
Tension wood induction by gravistimulation. Two-month-old poplar plantlets grown in the greenhouse were laid on the trays for 3 weeks, approximately 80 degrees relative to the vertical position. **(A)** Poplar plantlets of the WT, phbmt1 mutants, and PHBMT1-overexpression (OE) lines at the initial point of gravistimulation trial. **(B)** Poplar plantlets of the WT, phbmt1 mutants, and PHBMT1-OE lines at the end point of 3-week gravistimulation. From far to near are the WT, phbmt1 mutants g1-8 and g1-9, and overexpression lines OE1 and OE2, respectively. **(C)** Illustration of definition of the top stem, bottom stem, stem lift height and the stem secant binding angle of poplar plantlet with gravitropic stimulation.

For trees subjected to gravistimulation, 2-month-old hybrid aspen plantlets with similar initial growth were selected and laid on the tray (inclined ca. 80 degrees) for 3 weeks in the greenhouse (Figure 1A), which is sufficient to develop TW according to Gerttula et al. (2015). Each tray contains a whole set of *PHBMT1* knockout mutant, OE and WT control lines. After treatment, the trees were photo-recorded for stem bending angle calculation. Then, their bark was removed from the top (TW) and bottom (OW) quarter of the curved stem, and the differentiating xylem was harvested as described above, which was used for RNA preparation. The remaining TW and OW fractions were collected and used for cell wall preparation and cell wall composition analysis. The trees without gravistimulation serve as the control for NW, in which the quarter of stem (NW) at the same internodes as the bent trees were harvested.

### Measurement of Poplar Stem Morphology With Gravistimulation

The method described by Gerttula et al. (2015) was adopted for the measurements of the stem secant angle, lift angle, stem length, and lift height after gravitropic stimulation. After gravistimulation, the plantlets with curved stems were photo-recorded and their stem secant angle was measured using Image J as illustrated in Figure 1C. To minimize the experimental variations caused by potential additional environmental factors, the calculated secant bending angle of the transgenic plant was normalized to that of the corresponding WT control in each tray. The data were presented as the relative secant bending angle, i.e., relative secant bending angle = secant bending angle of transgenic line/secant bending angle of WT in the same tray. The stem length and lift height were also measured using Image J as illustrated in Figure 1C. We defined the point at the stem with the sharpest angle as the turning point for vertical growth; then the top portion of the stem from the tip to the turning point was defined as the top stem and the stem below the turning point was defined as bottom stem (Figure 1C). The ratio of top stem length/bottom stem length was calculated and used to describe the position of set-point-angle caused by the composite response to autotropism and gravitropism. The lift angle was measured using Image J as illustrated in Supplementary Figure 1A. The statistical analysis was performed using a Student’s *t*-test with two-tailed distribution and two-sample unequal variance.

### RNA Extraction and Real-Time Quantitative PCR Analysis for Gene Expression

Total RNAs were extracted from the collected poplar developing xylem using Spectrum Plant Total RNA Kit (Sigma-Aldrich, St. Louis, MO, United States) with on-column DNase digestion following the instructions of the manufacturer. Then, 1 μg total RNAs were reverse-transcribed to cDNA using Applied Biosystems High-Capacity cDNA Reverse Transcription kit (Thermo Fisher Scientific, Waltham, MA, United States) following the instructions of the manufacturer. The obtained cDNA was used for qRT-PCR reactions with the primers listed in Supplementary Table 1. The constitutively expressed actin gene was used as the reference gene with primer listed in Supplementary Table 1. qRT-PCR was performed on a C1000 Touch Thermal cycler (BioRad, Hercules, CA, United States) in a 10-μl volume containing 10 ng of cDNA, 4 pM of each primer, and 5 μl of SsoAdvanced Universal SYBR Green Master Mixture (BioRad, Hercules, CA, United States), according to the protocol of the manufacturer. The qRT-PCR program consisted of 3 min of initial denaturation at 94°C, followed by 40 cycles of 10 s at 94°C, 20 s at 58°C, 20 s at 72°C, and a final melting curve analysis. Three biological repeats were used for each tissue or genotype. Relative expression levels of the selected genes were presented using the 2^–Δ^ ^Δ^ ^Ct^ method (Livak and Schmittgen, 2001).

### Preparation of Extractive-Free Cell Wall Residues and Wall-Bound Phenolics Analysis

The harvested poplar woody materials were dried at 40°C to the constant weight and ground with a Wiley mill. The milled woody powders after passing through a 60-mm mesh sieve were extracted with 70% ethanol at 65°C for 3 h. This extraction process was repeated three times. The residues were further extracted with chloroform/methanol (1:1, v/v) three times and treated with acetone at room temperature overnight. The residues were dried at 40°C and then extracted by 90% dimethyl sulfoxide (DMSO) overnight to de-starch the cell walls. The DMSO extraction was repeated two times, and the residues were further washed with 70% ethanol and acetone. The extractive-free residues were dried at room temperature and were referred to as de-starched extractive-free cell wall residues (CWRs).

The wall-bound phenolics were released from ca. 5 mg CWRs with 0.5 ml 2N NaOH in the dark at 37°C for 16 h. After acidification of the reaction with 0.3 ml 4N HCl, 10 μl 1 mM chrysin was added as internal standard. The hydrolysates were then partitioned with 400 μl water-saturated ethyl acetate three times. The extracts were pooled and dried under vacuum then, dissolved in 100 μl 80% methanol. Then, 3 μl samples were injected for ultraperformance liquid chromatography-mass spectrometry (UPLC-MS) analysis with a Dionex Ultimate 3000 UHPLC system hyphenated with a Thermo Scientific Q-Exactive Plus mass spectrometer. The extract was resolved chromatographically through a UHPLC C18 column (Luna, 150 mm × 2.1 mm, 1.6 μm, Phenomenex, CA, United States) with a mobile phase of water containing 0.1% acetic acid (solvent A) and 100% acetonitrile containing 0.1% acetic acid (solvent B) at room temperature with a flow rate of 0.1 ml min^–1^. The elution process was set with a gradient of mobile phase B in A as: 0–1 min, 5–30% B; 1–19 min, 30–80% B; 19–20 min, 80–99% B; 20–22 min, 99% B; 22–23 min, and 99–5% B. Full mass scan was set in the range of *m/z* 100–1,500. The mass spectrometer parameters were as follows: sheath gas flow rate 40 units (negative mode); aux gas unit flow rate 10; the capillary temperature at 350°C; aux gas heater temperature at 250°C, spray voltage 3.5 kV; S lens RF level at 60. Peaks were identified by comparing with the authentic standards based on their retention times, UV spectra and mass spectra. The amount of the wall-bound metabolites was quantified based on their peak area of UV absorbance at 280 nm, with standard curves of authentic standards.

### Lignin Composition Analysis

The Thioacidolysis method (Lapierre et al., 1986) was used to determine and quantify lignin composition with minor modification. The sum of thioacidolytic monomers G and S was used as a proxy for lignin content in this study. Briefly, for each sample, 10 mg of CWRs were mixed with 1 ml of the freshly prepared reaction mixture [2.5% boron trifluoride etherate and 10% ethanethiol in distilled dioxane (v/v)] in a 7 ml glass vial and flushed with N2 gas. Then, the vial was tightly sealed and heated at 100°C for 4 h with periodic agitation. The reaction was stopped by placing on ice for 15 min and then its pH value was brought to 3–4 by using 0.4 M sodium bicarbonate. Furthermore, 2 ml water was then added to the reaction. Meanwhile, 1 mg tetracosane (dissolved in 1 ml methylene chloride) was added to each vial as an internal standard. The vial was recapped, vortexed, and allowed to settle for more than half-hour until phase separation of the solution occurred. An aliquot (1.5 ml) of the organic phase was taken and passed through a Pasteur pipette packed with an inch of anhydrous sodium sulfate. The filtrate was then evaporated to dryness and resuspended in 0.5 ml of methylene chloride. The samples (50 ml) were then dried and derivatized with pyridine and N-methyl-N-(trimethylsilyl) trifluoroacetamide (Sigma) at room temperature for 5 h. Quantifying the corresponding monomers was accomplished *via* a gas chromatography-flame ionization detector (FID) on a GC instrument (Agilent 7890A, CA, United States) implemented on a HP-5 ms fused silica capillary column (30 m × 0.25 mm, 0.25-μm film thickness, Agilent) with following settings: helium as carrier gas at 1.5 ml min^–1^, an inlet pressure of 21.391 psi, heater temperature 250°C, total flow 12 ml min^–1^, septum purge flow 3 ml min^–1^, split ratio 5:1, and oven temperature programmed from 130°C ramped at 10°C min^–1^ to 180°C, then ramped at 3°C min^–1^ to 255°C, held 5 min. FID was accomplished with heater 300°C, H2 flow 35 ml min^–1^, air flow 400 ml min^–1^, and makeup flow (He) 5 ml min^–1^. The quantification for syringyl and guaiacyl lignin monomers was based on the relative peak area to the tetracosane internal standard with a response factor of 1.5 according to the described (Lapierre et al., 1986).

### Crystalline Cellulose Quantification

Crystalline cellulose content was determined *via* the colorimetric Updegraff method as previously described (Foster et al., 2010). Briefly, ∼1 mg CWRs were mixed with 250 μl of 2 M trifluoroacetic acid (TFA) and heated at 121°C for 90 min. The TFA was evaporated under a stream of air at 40°C and the pellet was washed with 300 μl of 2-propanol and dried under a stream of air, three times. Then, the pellet was suspended in 500 μl of water with a vortex application. After centrifugation, the supernatant was discarded, and the pellet was vacuum dried at 50°C. The dried pellet was treated with 1 ml of Updegraff reagent [acetic acid: Nitric acid: Water, 8:1:2 (v/v)] at 100°C for 30 min. The samples were allowed to cool at room temperature and spun down at 21,000 g for 10 min, resulting in a pellet consisting primarily of crystalline cellulose. The supernatant was removed, and the pellet was washed once with 1 ml of water and four times with 1.5 ml of acetone and pelleted at 21,000 g for 10 min each time. After drying at room temperature, the crystalline cellulose was then hydrolyzed with 175 μl of 72% (w/w) sulfuric acid for 1 h at room temperature under 250 rpm agitation. After hydrolysis, 825 μl of water was added, and the samples were spun down at 21,000 g for 5 min. The remaining solution was used for glucose determination using the anthrone assay with a glucose standard curve. The absorbance was measured at room temperature at 625 nm.

## Results

### Mechanical Stress and Gravistimulation Induce *PHBMT1* Expression

**FIGURE 2:**
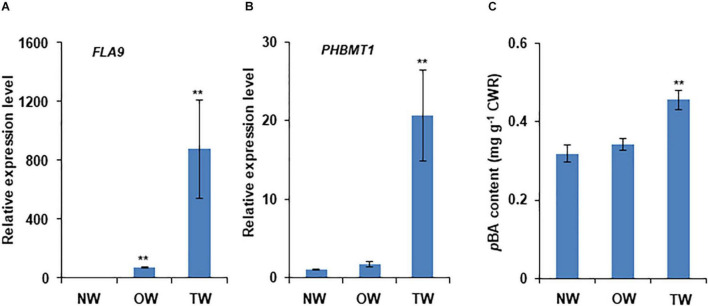
Induction of *PHBMT1* gene expression and accumulation of wall-bound *p*-hydroxybenzoates in poplar tension wood upon mechanical bending. One-year-old hybrid poplar tree was subjected to mechanical bending for 10 days. **(A)** Relative expression levels of tension wood marker gene *FLA9* in normal wood (NW), opposite wood (OW), and tension wood (TW) detected *via* real-time quantitative PCR (qRT-PCR). The expression level in NW is set as 1 and the actin gene as the reference. **(B)** Relative expression levels of *PHBMT1* in NW, OW, and TW. The expression level in NW is set as 1 and the actin gene as the reference. **(C)** Content of the alkaline-released wall-bound *p*-hydroxybenzoates (*p*BAs) in the developing xylem of NW, OW, and TW. CWR, cell wall residue. Data in **(A–C)** represent the mean ± SD of three biological repeats. Asterisk indicates significant difference compared to the corresponding NW, **P < 0.01 (Student’s *t*-test).

Our recent study revealed that *PHBMT1* broadly expresses in the root, stem, leaf, and apical bud of *P. trichocarpa* with higher expression levels in root and stem xylem under normal growth conditions (Zhao et al., 2021). To further explore *PHBMT1* expression under environmental stresses, we examined its transcript abundance in the trees under mechanical stress and/or gravistimulation. Mechanically bending the stems or displacing the trees with respect to gravity can induce poplar TW formation (Andersson-Gunneras et al., 2006; Foston et al., 2011; Gerttula et al., 2015). We adopted both approaches in the study of *PHBMT1* expression. Since extremely high transcript abundance of fasciclin-like arabinogalactan (FLA) gene is a known characteristic of TW, *FLAs* are routinely used as the molecular markers of TW development (Lafarguette et al., 2004; Andersson-Gunneras et al., 2006; Gerttula et al., 2015). Therefore, *FLA9* was included in our analysis. When 1-year-old hybrid aspen stems were bent for 10 days, qRT-PCR analysis with RNAs from the scrapped developing xylem revealed that the *FLA9* transcripts were elevated approximately 800-fold in the developing xylem of the upper side of the bent stem, compared with that in the lower side of the bent stem, indicating the formation of TW at the upper side of the treated stem (Figure 2A). When the expression level of *PHBMT1* was examined, similar to *FLA9*, its transcript abundance showed about 20 times increase in the upper side of the bent stem (i.e., TW), relative to that in the lower side of the bent stem (OW) and the trees without bending (NW) (Figure 2B). Subsequently, the alcohol extractive-free CWRs were prepared from the collected developing xylem of the upper and lower sides of bent stems and the stems without bending, followed by mild alkaline hydrolysis to release the wall-bound *p*BAs, i.e., the lignin-bound *p*BAs, since only lignin fraction in the poplar cell walls is acylated with *p*BAs (Lu et al., 2004; Zhao et al., 2021). We found that the *p*BAs released from the developing xylem of TW were increased by 43.5%, compared with normal or OW (Figure 2C). These data indicate that *PHBMT1* expression and the accumulation of lignin-bound *p*BAs are induced by mechanical stress and are accompanied by the formation of TW in poplar.

**FIGURE 3:**
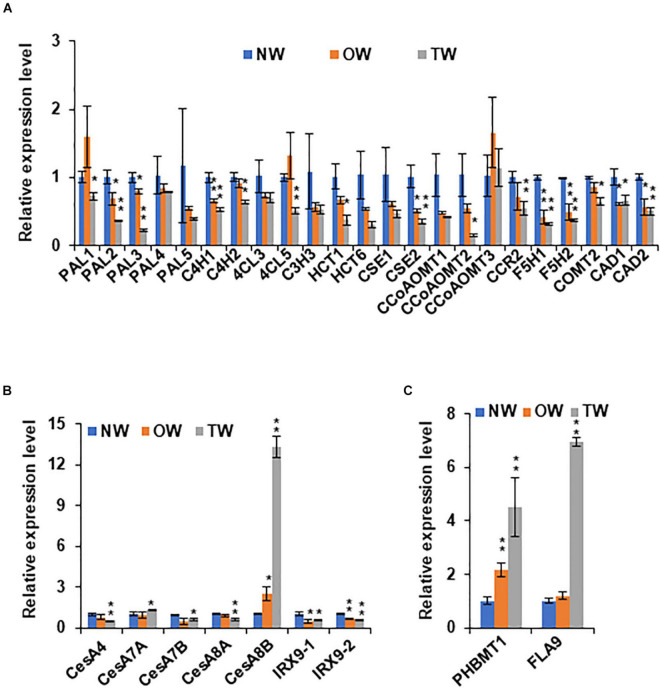
The expression of secondary cell wall synthesis related genes in poplar stem under gravitropic stimulation. **(A)** Relative expression levels of monolignol biosynthetic genes in NW, OW, and TW of hybrid aspen plantlets. **(B)** Relative expression levels of secondary cell wall related cellulose synthase (CesA) genes and xylosyltransferase genes *IRX9-1* and *IRX9-2* in NW, OW, and TW of hybrid aspen plantlets. **(C)** Relative expression levels of *PHBMT1* and *FLA9*, a marker gene for TW. Gene expression in NW was set as 1. Data in **(A–C)** represent the mean ± SD of three biological repeats. Asterisk indicates significant difference compared with the corresponding normal wood, *P < 0.05, **P < 0.01 (Student’s *t*-test).

To further examine and verify the involvement of poplar *PHBMT1* and lignin *p*BAs accumulation in response to mechanical stress, we adopted the gravistimulation method as described by Gerttula et al. (2015) to treat poplar plantlets. In the treatment, we had included the *PHBMT1* knock-out and OE lines besides the WT plants, aiming to compare their potential different response behaviors to gravitropic stimulation (as shown in section “Results”). About 2-month-old hybrid aspen plantlets were subjected to 80 degrees of gravistimulation (i.e., laid down near on the ground) for 3 weeks (Figure 1A). By the end of treatment, the poplar plantlets displayed an obvious gravitropic response with their stems pulled upward from the original nearly horizontal position with an obvious curvature (Figure 1B). The RNAs were then prepared from the developing xylems of both the upper and lower sides of stem fractions at the curved region. The qRT-PCR analyses were performed for a whole set of previously defined poplar xylem-expressing monolignol biosynthesis genes, the secondary cell wall cellulose synthase (*CESA*) genes, *CESA4, 7*, and *8*, and the genes involved in hemicellulose biosynthesis, *IRX9-1* and *IRX9-2* (Shi et al., 2017). *FLA9* was included as a TW marker. As delineated in Figure 3A, the expression of several monolignol biosynthetic homologous genes, such as *PAL3*, *C4H2*, *CSE2*, and *CcoAOMT2*, was significantly downregulated in the gravistimulation-induced TW, compared with that either in NW or OW. Interestingly, among the defined secondary cell wall *CESAs*, only *CESA8B* showed dramatic induction. Its transcript level raised more than 12-fold in TW compared with that in NW, implicating that *CESA8B* might play a primary role for gelatinous cellulose fiber synthesis in TW (Figure 3B). Consistent with the previous report that hemicelluloses were decreased in poplar TW (Hedenstrom et al., 2009; Groover, 2016), the genes encoding xylosyltransferase for the formation of xylan backbone, *IRX9-1* and *9-2*, were downregulated in TW after gravitropic stimulation. Similar to the observation in poplars with bending treatment (Figure 3) and aligned well with the induction of *FLA9* gene expression, the expression level of *PHBMT1* in plantlets upon gravitropic stimulation also displayed significant elevation in TW, compared with that in OW and NW (Figure 3C), demonstrating a strong response of *PHBMT1* to gravistimulation in poplar plantlets along with TW formation. Interestingly, the expression of both *CESA8B* and *PHBMT1* was also found to be slightly induced in the OW (Figures 3B,C).

### Altering *PHBMT*1 Expression Affects Poplar Gravitropic Response Behaviors

**FIGURE 4:**
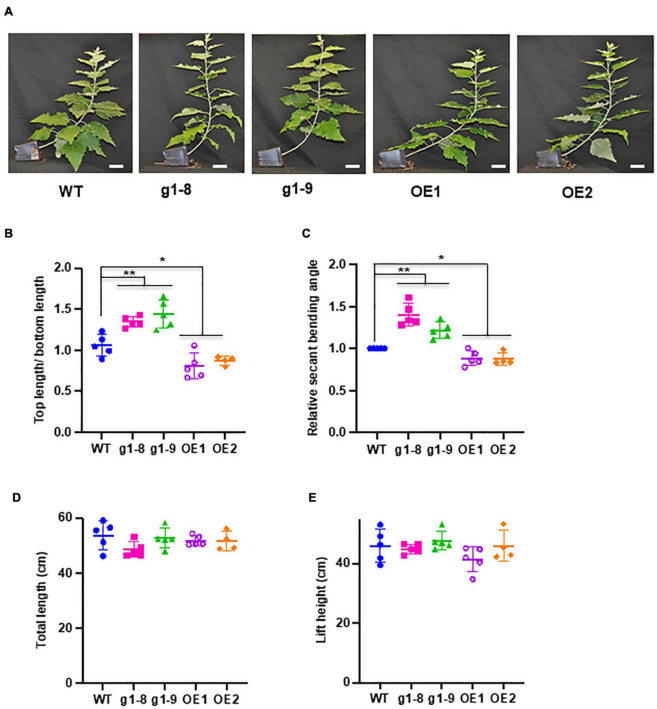
Response of *PHBMT1* knockout mutant and OE lines to gravitropic stimulation. **(A)** Morphology of hybrid aspen plantlets at the end point of 3-week gravitropic stimulation to the wild type (WT), *phbmt1* knockout (g1-8, g1-9), and OE lines (OE1 and OE2). Bar = 5 cm. **(B)** The ratio of the top (near) vertical stem length/bottom inclined curving stem length of the WT, *phbmt1* mutant (g1-8 and g1-9), and OE plantlets (OE1 and OE2) under gravitropic stimulation. **(C)** The calculated relative stem secant binding angle of the WT, *phbmt1* (g1-8 and g1-9), and OE plantlets (OE1 and OE2) under gravistimulation. The stem secant binding angle of each corresponding WT was set as 1. **(D,E)** The total stem length **(D)** and the lift height **(E)** of the WT, *phbmt1* knockout (g1-8 and g1-9), and OE plantlets (OE1 and OE2) at the end point of 3-week gravitropic stimulation. Data represent means ± SD of four or five biological repeats. Asterisk indicates significant difference compared with the corresponding WT, *P < 0.05, **P < 0.01 (Student’s *t*-test).

With the recognition of significant induction of *PHBMT1* expression in poplar with gravistimulation, we intended to further decode the physiological significance of *PHBMT1* and the PHBMT1-mediatd monolignol (eventually lignin) *p*-hydroxybenzoylation on plant growth in response to gravistimulation. As depicted in Figure 1, 2-month-old WT, *PHBMT1* knockout, and OE hybrid aspens were subjected to gravistimulation for 3 weeks, and the posture/morphology of the plantlets was monitored. Previously, we demonstrated that knocking out *PHBMT1* nearly completely depleted the accumulation of *p*BAs in the lignin of the poplar stem; whereas the *PHBMT1* OE lines showed up to a 48% increase of the alkaline-releasable *p*BAs relative to the WT (Zhao et al., 2021). When the plantlets received gravistimulation for 3 weeks, the WT and transgenic plantlets displayed discernible phenotypic differences (Figure 4A). The *PHBMT1* knockout plants appeared to have a more enhanced upward growth in response to the gravity stimulation. Their originally inclined stems were pulled upward more sharply and obviously; the verticality of the originally inclined stem apparently occurred at the lower/more basal internodes, which yielded a relatively larger portion of vertical trunk and shorter inclined trunk, compared with the WT (Figure 4A). By contrast, the stem curving of the *PHBMT1* OE lines took place more at the higher/apical internodes, which gave rise to a larger portion of inclined basal stem without curving, in comparison with the WT and the knockout mutant lines (Figure 4A). Measuring the position where the inclined/curved stem transits to the (near) vertical stem, which was represented with the ratio of the top (near) vertical stem length over the bottom inclined/curved stem length (Figure 1C), it quantitatively confirmed the clear differences among the WT, *PHBMT1* knockout, and OE lines (Figure 4B). Determining stem secant bending angle as depicted in Figure 2, revealed 23∼40% increases of the stem binding angle in the *PHBMT1* knockout plants g1-8 and g1-9, in comparison with that in the WT (Figure 4C). By contrast, the calculated secant bending angle of the *PHBMT1* OE plants was decreased about 12%, compared with the WT (Figure 4C). Considering that trunk length/height could influence the calculation of stem secant bending angle, we further measured the trunk length and stem lift height (depicted in Figure 1C). The data showed no significant difference among the WT, *PHBMT1* knockout, and OE plants (Figures 4D,E), confirming that disturbing *PHBMT1* expression does not affect the plant height as we previously reported (Zhao et al., 2021). Furthermore, to avoid any potential influence of the stem height variations on the calculation of secant binding angle, as an alternative, we determined the basal stem lift angle as depicted in Supplementary Figure 1A. Consistent with the calculated secant bending angle, the basal stem lift angle of the *PHBMT1* knockout plants was larger than that of the WT, while it was smaller for the *PHBMT1* OE plants (Supplementary Figure 1B). These morphological observations and quantitation indicate that alteration of *PHBMT*1 expression exerts substantial influence on the poplar gravitropic response behaviors.

### Accumulation of Wall-Bound *p*BAs Negatively Correlates With Poplar Gravitropic Response

**FIGURE 5:**
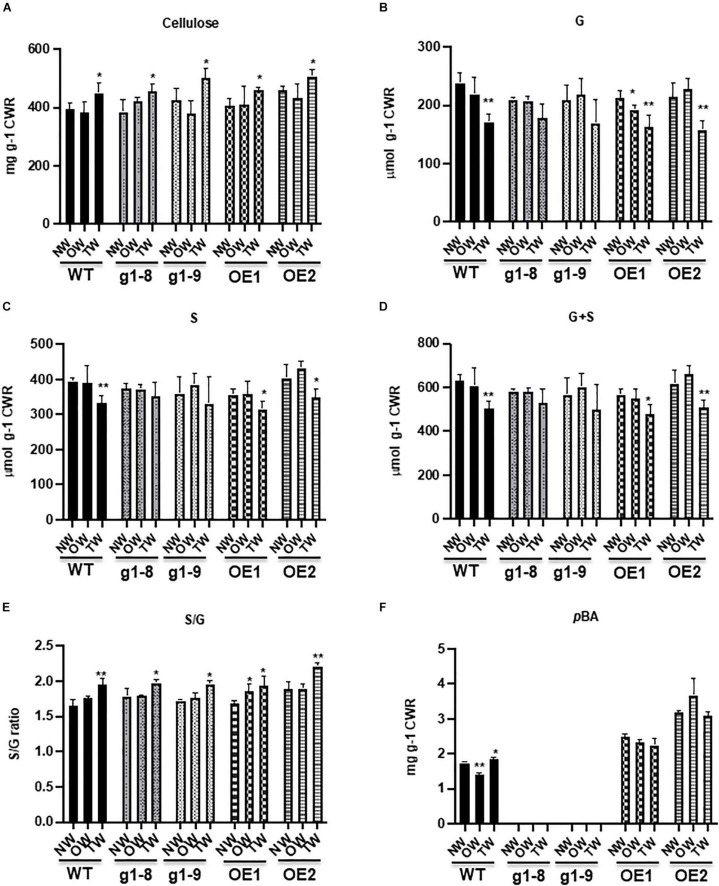
Accumulation of crystalline cellulose, lignin, and wall-bound *p*-hydroxybenzoates in the WT, *phbmt1* mutant, and OE lines of hybrid aspen. Content of crystalline cellulose **(A)**, thioacidolysis-cleaved G **(B)** and S **(C)** monomers, the total G + S monomers **(D)**, the S/G ratio **(E)**, and the alkaline-released wall-bound *p*-hydroxybenzoates **(F)** in NW, OW, and TW of the WT, *phbmt1* knockout lines (g1-8 and g1-9), and OE lines (OE1 and OE2). CWR, cell wall residue. Data in **(A–F)** represent the mean ± SD of four biological repeats. Asterisk indicates significant difference compared with the corresponding normal wood, *P < 0.05, **P < 0.01 (Student’s *t*-test).

To gain further insight into the potential cell wall compositional alterations of poplars under gravistimulation, we quantified the content or composition of cell wall cellulose, lignin, and *p*BAs in normal, tension, and opposite wood of the WT, *PHBMT1* knockout mutant, and OE lines, respectively. As expected, upon gravistimulation, cellulose synthesis in the WT was significantly enhanced in TW. The crystalline cellulose content was increased 14% compared with that in NW, and 17% relative to that in OW (Figure 5A), exhibiting a typic characteristic of TW formation. However, the levels of both G- and S-lignin monomers released *via* diagnostic thioacidolysis that specifically cleaves β-*O*-4 aryl ether linkages of lignin polymer, showed a significant reduction in the cell walls of TW. Moreover, the G-lignin monomers were reduced more severely than the S-lignin monomers, with a 28% decrease in G but a 16% decrease in S compared with their corresponding levels in NW (Figures 5B,C). As such, an increased S/G ratio occurred in the WT TW lignin (Figure 5E), indictive of a relatively higher abundance of S-lignin in the gravistimulation-induced reaction wood, even though the sum of G + S monomers, representing total lignin content, was substantially reduced in that wood (Figure 5D). Similar to the poplar trees imposed with mechanical bending, gravistimulation induced the accumulation of lignin-bound *p*BAs in TW; meanwhile, decreased its level in OW compared with the WT NW. The content of alkaline-released *p*BAs in TW of the WT was about 6% higher than that in NW and approximately 32% higher compared with that in OW (Figure 5F), which leads to an asymmetric distribution of *p*BA esters in the wood under gravistimulation.

Knocking out *PHBMT1* in hybrid aspen not only nearly completely abolished *p*BAs in NW, as we reported recently (Zhao et al., 2021), but also diminished *p*BAs accumulation in TW and OW (Figure 5F), suggesting that PHBMT1 is not only the prime enzyme responsible for the accumulation of poplar stem lignin *p*BAs in normal growth condition but also the one under gravitropic stress. Conversely, the OE of *PHBMT1* resulted in the overproduction of *p*BAs in the cell walls of both TW and OW of the transgenic lines; the accumulation levels of *p*BAs were essentially the same in TW, OW, and NW of the OE transgenic lines, which were about 85% higher than that in NW of the WT (Figure 5F).

Consistent with our previous report on the total lignin content determined by the acetyl bromide method (Zhao et al., 2021), disturbing *PHBMT1* expression (knockout or OE) resulted in a little or no significant alteration of lignin content (represented with the amount of G + S) in NW of both the *PHBMT1* knockout and OE transgenic lines, compared with that in the WT NW (Figure 5D). Previously, our NMR study on the cellulolytic enzyme lignins from both *PHBMT1* knockout and OE plants also suggested that neither knockout nor OE of *PHBMT1* severely affected the distribution of the major intermonomeric linkage types, such as β-aryl ether, phenylcoumaran, resinol, and spirodienone units, in the lignin polymer (Zhao et al., 2021). Similar to that occurred in the WT, gravistimulation caused the reduction of both G- and S-lignin monomers in TW of *PHBMT1* knockout lines (g1-8 and g1-9) (although the reduction appeared no statistical significance due to the large variation of the data), and of the *PHBMT1* OE lines, in comparison with their corresponding NW (Figures 5B,C). Similarly, the S/G ratio was increased in TW of both the *PHBMT1* knockout and OE lines, indictive of the increase in the relative abundance of S-lignin subunits as that observed in the WT TW (Figure 5E).

*PHBMT1* knockout or OE, in general, did not affect the cellulose fiber deposition in their NW cell walls, compared with the WT, except for one OE line (OE2) that showed a slightly higher content of cellulose fibers in its NW (Figure 5A). Similar to the WT plants, under gravistimulation, the cellulose deposition in TW of both *PHBMT1* knockout and OE lines was significantly increased, compared with their corresponding NW. The final amounts of crystalline cellulose fibers in TW of the knockout and OE lines were at a similar level as that in TW of the WT, suggesting that altering *PHBMT1* expression and changing in lignin-bound *p*BA accumulation does not affect the inducible accumulation of crystalline celluloses pertaining to the development of TW. Overall, the cell wall chemical changes, such as crystalline cellulose fibers, total cleavable lignin monomers (S + G), and its S/G ratio in the TW of *PHBMT1* knockout and OE lines were similar to those occurred in the WT TW.

## Discussion

In response to gravity, angiosperm trees approach a gravitropic response accompanied by the production of TW. The biphasic process of gravitropic response programs with the first phase of re-orientation of displaced organs (i.e., gravitropism), followed by autotropic straightening phase (autotropism) (Stankovic et al., 1998a,b; Bastien et al., 2013). The latter was conceived of as a counter-reaction to gravitropic reorientation, thus also called the decurving process (Stankovic et al., 1998b). A composite response of gravitropism and autotropism results in the final set-point angle of an organ.

Gravitropic movements have been observed in a large range of herbaceous species and woody plants. It relies on the asymmetric production of reaction wood (TW in angiosperms). Differential maturation between the two sides of the organ in zones where elongation is completed but radial growth still active results in the occurrence of stem curvature. Therefore, TW production in angiosperm is part of a corrective growth mechanism that generates tensile stress on the upper part of the tilted stem/branch, allowing the tree to continuously adjust its position to the multiple disturbances (Ghislain and Clair, 2017). Production of highly crystalline cellulose fibers in TW is known critical for generating tensile growth stress to sustain organ re-orientation (Okuyama et al., 1994; Yamamoto, 1998; Yoshida et al., 2002; Groover, 2016). In contrast to the relatively well understanding of the importance of cellulose microfiber in gravitropic movement and the knowledge on lignin accumulation in gymnosperm compression wood (the wood produced on the lower part of the tilted stem in gymnosperms, which generates compressive stress), so far there is limited information regarding the physiological or mechanical roles of lignin and lignin modification in angiosperm TW in regulating the gravitropic movement. Particularly, it is not clear whether discrete lignin deposition and/or modification can affect the function of TW fibers. The previous studies indicate that there is a decrease in lignin content in TW accompanied by an increase of the S/G ratio in the secondary cell wall (Pilate et al., 2004). This type of chemical shift is well verified in several previous NMR spectroscopy-based chemical studies on poplar TW (Hedenstrom et al., 2009; Foston et al., 2011; Al-Haddad et al., 2013).

Consistent with the previous studies, our chemical analysis confirms that when poplars are imposed to gravistimulation, the crystalline cellulose content in the upper side of the tilted stem was increased more than 14% compared with its NW or OW, whereas the lignin content, represented with the summed G + S monomers in this study, was decreased up to 20%, meanwhile the S/G ratio showed a significant increase in TW fraction (Figure 5). Consistently, the expression of the TW marker gene *FLA9* and secondary cell wall related *CESA* genes was markedly induced, while a set of monolignol biosynthetic genes were downregulated (Figure 3). These data suggest that the poplar plantlets in our experimental system have been triggered for a typic gravitropic response and TW formation. While both G and S lignin subunits are decreased in TW, upon gravistimulation or mechanical bending, the lignin-bound *p*BAs are significantly increased (Figures 2, 5), which results in an asymmetric distribution of the *p*-hydroxybenzoylated lignin in the tilted stem. This observation is consistent with the previous chemical analysis that a higher amount of *p*BAs occurred in poplar TW (Hedenstrom et al., 2009; Foston et al., 2011; Goacher et al., 2021).

Accompanied with the elevation of lignin-bound *p*BAs is the significant induction of the expression of the *PHBMT1* gene in TW (Figures 2, 3), the gene encodes a BAHD family of acyltransferase that preferentially conjugates *p*-hydroxybenzoate to S-lignin monomer sinapyl alcohol. When the conjugates incorporate into lignin polymer, it yields the *p*-hydroxybenzoylated lignin (Zhao et al., 2021). Interestingly, disruption of the gene by CRISPR/Cas9-mediated gene editing depleted the *p*BA accumulation not only in the cell walls of NW of the WT poplar but also prevented the inducible accumulation of *p*BAs in the TW of the trees under gravistimulation (Figure 5), which strongly suggests that PHBMT1 is not only the prime enzyme responsible for the accumulation of lignin-bound *p*BAs in the wood formed under normal growth condition but also the sole enzyme for the inducible *p*BA accumulation in cell wall under gravistimulation or mechanical stress, although *PHBMT1* has several close homologous genes in *Populus* genome (Zhao et al., 2021).

The significant induction of *PHBMT1* expression and the enhanced accumulation of *p*BAs in TW of the plants upon mechanical bending and gravistimulation suggest that the lignin modification with *p*BAs might be involved in the mechanical stress/gravitropic response. This notion is further evident with the differential response behaviors and growth phenotypes of the *PHBMT1* knockout and OE trees under gravistimulation. The larger secant bending angle (and the stem lift angle) and more obvious upward curving at the basal internodes were observed in the *PHBMT1* knockout plants, where the lignin-bound *p*BAs are eliminated (Figure 4 and Supplementary Figure 1); by contrast, when *p*BAs were hyper-accumulated in the stem cell walls of the *PHBMT1* OE plants, smaller secant bending angle was determined and the stem upward curving occurred at the apical internodes (Figure 4). It is important to note that except for the alteration in *p*BA quantity in the *PHBMT1* knockout and OE poplars, disturbing *PHBMT1* expression, in general, did not cause significant alterations in other major cell wall components, such as accumulation of cellulose, and lignin content, composition, and intramolecular cross-linkages, which were revealed in our previous (Zhao et al., 2021) and present studies (Figure 5). Furthermore, under gravistimulation, the stress-induced cell wall compositional shifts, such as the increase in crystalline cellulose fibers, a decrease of lignin content, and increase of lignin S/G ratio, in TW of the *PHBMT1* knockout and OE plants are in large extent the same has occurred in the WT (Figure 5). Taken together of those data, it demonstrates that the alteration of lignin-bound *p*BAs (due to the disturbance of *PHBMT1* expression) is the major cause for the differential gravitropic responses of the transgenic poplars. Therefore, the PHBMT1-mediated monolignol *p*-hydroxybenzoylation and the subsequent accumulation of lignin-bound *p*BAs play observable roles in the regulation of poplar gravitropic response to sustain poplar growth and the posture of trees.

The exact molecular mechanism on how lignin-bound *p*BAs affect the gravitropic response is currently not certain. One possibility is that the enhanced *p*BA accumulation in TW somehow compromises the gravitropism, i.e., the upward curving activity of the displaced trees. This could be caused presumably by the change in lignin physicochemical property along with the alteration of *p*-hydroxybenzoylation. Alternatively, and more likely, it might affect the autotropic straightening, i.e., decurving process, one of the biphasic processes in gravitropic response that imposes negative regulation to the organs undergoing gravitropic reorientation. This assumption reflects the observation of the clear difference of the position of the set-point angle that leads to the return of the inclined stem to the upward growth in the WT, the *PHBMT1* knockout, and OE plants (Figure 4, represented with the ratio of top stem over bottom stem). The shoot verticality of the inclined *PHBMT1* knockout plants started at more basal internodes, showing a stronger tendency in recovering the stems to the vertical posture from their original horizontal displacement. This contrasts to the phenotypic alteration in the *PHBMT1* OE plants that hyper-accumulate lignin-bound *p*BAs, where gravitropic curving/vertical growth took place largely at the apical internodes that leads to a larger portion of the inclined basal stem (Figure 4), likely suggesting an enhanced autotropic strengthening.

Our assumption of lignin-bound *p*BAs involved in the regulation of gravitropism or autotropism process is further supported with the observation of the unique growth phenotype of the *PHBMT1* knockout poplars grown in a semi-controlled greenhouse condition. As it was briefly described in our recent publication (Zhao et al., 2021) and as depicted specifically in Supplementary Figure 2 in the present study, about a quarter of the *PHBMT1* knockout poplar trees grown in the greenhouse had developed discernible S-shape/twisted trunks (Supplementary Figure 2), distinct from the straight upright growth of the WT controls. In those 6∼7-month-old trees, the lignin content, composition and structure, and stem biomass yield are at the same level as those in the WT control (Zhao et al., 2021). Therefore, it is most likely that the defect in *PHBMT1* and lignin-bound *p*BA accumulation in those trees impairs the well-balanced and controlled intrinsic gravitropism and autotropism regulatory mechanism, which leads to an over-reaction to gravity or unexpected mechanical stresses enforced by the environmental factors. The reason that only a portion of the knockout poplar population displayed the described phenotype is probably due to the unexpected, non-uniform environmental stimulation, e.g., wind from exhaust fans on the walls of a semi-controlled greenhouse condition.

The wall-bound *p*BAs have been demonstrated to primarily distribute in the fibers of the poplar xylem (Goacher et al., 2021), seemingly implicating their potential role in imparting fiber wall mechanical strength. It remains to determine whether the inducible accumulation of *p*BAs under gravistimulation is also restricted to the xylem fiber cells and, more specifically, if they occur within the G-layer of TW fiber wall and alter the TW contractile and mechanical properties. Although lignification of G-layer of TW has been detected in many species (Ghislain and Clair, 2017), whether the G-layer of poplar TW fiber is lignified is a matter of debate, and the evidence on the existence of lignified G- layer is ambiguous. The earlier study *via* transmission electron microscopy (TEM)-immunogold imaging with antibodies for guaiacyl and syringyl lignin epitopes suggested a low or trace amount of lignin detected in the G-layer of TW of poplar species and a shift in lignin composition with an increase in the relative abundance of S-lignin units into the G-layer with respect to the S2 wall layer (Joseleau et al., 2004; Pilate et al., 2004). Whereas, the later label-free chemical imaging *via* UV microscopy, TEM, confocal Raman microscopy, or TEM, confocal Raman microscopy, or time-of-flight secondary ion mass spectrometry (ToF-SIMS) revealed almost no lignin in the G-layer of poplar TW (Gierlinger and Schwanninger, 2006; Jung et al., 2012; Yoshinaga et al., 2012). The distribution of pBAs in the TW remains to be further explored. In addition, lignin acylation is encountered in different plant species with different types of carboxylic groups. In particular, the lignin-bound *p*CAs as pendants are prevalent in Commelinid grass walls. It is interesting to explore whether those lignin modifications also play a similar role in gravitropic response as observed for lignin *p*BAs in poplar species.

Taken together, our study reveals a unique adaptive mechanism of poplar species in response to the mechanical stress/gravistimulation, in which the enhanced *PHBMT1* expression and S-lignin *p*BA accumulation appear to participate in the regulation of poplar gravitropic response, presumably by affecting gravitropic re-orientation and autotropic straightening to coordinate with the production of crystalline cellulose fibers in TW to control the deformation of poplar wood and to sustain poplar posture.

## Data Availability Statement

The original contributions presented in the study are included in the article/Supplementary Material, further inquiries can be directed to the corresponding author/s.

## Author Contributions

C-JL conceived the research plan and wrote the manuscript. X-HY initiated a mechanical stress experiment. YZ conducted the experiments. C-JL and YZ designed the experiments, analyzed, and interpreted data. All authors contributed to preparing the manuscript and approved the submitted version.

## Conflict of Interest

The authors declare that the research was conducted in the absence of any commercial or financial relationships that could be construed as a potential conflict of interest.

## Publisher’s Note

All claims expressed in this article are solely those of the authors and do not necessarily represent those of their affiliated organizations, or those of the publisher, the editors and the reviewers. Any product that may be evaluated in this article, or claim that may be made by its manufacturer, is not guaranteed or endorsed by the publisher.
