# Supplementary material for: The Inducible Accumulation of Cell Wall-Bound p-Hydroxybenzoates Is Involved in the Regulation of Gravitropic Response of Poplar
Source: Front Plant Sci. 2021 Dec 14;12:755576. doi: 10.3389/fpls.2021.755576 (PMC8712735; doi:10.3389/fpls.2021.755576)
Supplement: Supplementary file 1 [file Table_1.DOCX]

**Supplemental Table 1. The primers used for qRT-PCR analysis in this study**

| Primer name | Sequence (5'-3') | Reference Gene ID |
| --- | --- | --- |
| PHBMT1-F | GTGATGCCATTGGCCTAGTT | Potri.001G448000 |
| PHBMT1-R | TCGCCATATTCGTTGTGTGT |  |
| FLA9-F | TTCTTATCCTTTGTCCGCCTG | Potri.009G012100 |
| FLA9-R | GGTTACTTAGAGTCTGGAAATCGG |  |
| PAL1-F | TGTTTCCAATCCCTTCTATGTT | Potri.006G126800 |
| PAL1-R | CATGCATCAATAGCTGTGTGAG |  |
| PAL2-F | CTTGGAAGCCATCACCAAGTTGCTC | Potri.008G038200 |
| PAL2-R | GTTTCTCCATTGGGTCCCACG |  |
| PAL3-F | ATGTACGTTTGGCTATTGCATC | Potri.016G091100 |
| PAL3-R | CCCTTGAAGCCATAATCCAAAC |  |
| PAL4-F | TAAACAAGACCAATATGCTCTT | Potri.010G224100 |
| PAL4-R | GACAGAGTTGATTTCTCGTTCG |  |
| PAL5-F | CGAACGAGAAATCAACTCTGTC | Potri.010G224200 |
| PAL5-R | GAGAATTGTGCGAACATGAGTT |  |
| C4H1-F | AGTGCGCCATAGACCATATCCTG | Potri.013G157900 |
| C4H1-R | ATACCAGCGACGTTGATGTTCTCA |  |
| C4H2-F | ACTTCCCTTTTGTTTTGTTTCG | Potri.019G130700 |
| C4H2-R | TGAGAATTCACATGGTCTAGGG |  |
| C3H3-F | GTGAGGACCGAGGACCAAATAA | Potri.006G033300 |
| C3H3-R | GATGTTGGAGTCTACCCCAATG |  |
| 4CL3-F | GAGAAGTTCCTGTTGCATTTGT | Potri.001G036900 |
| 4CL3-R | CACCTGCATCACCATGATCATG |  |
| 4CL5-F | GTCACCGAAGAAGAAATTATGC | Potri.003G188500 |
| 4CL5-R | GAATGGCTTCTACGAAGAACAC |  |
| HCT1-F | ATCAGCATGTAAGGCACGCGG | Potri.003G183900 |
| HCT1-R | TGCCAAAGTAACCAGGTGGAAGTGT |  |
| HCT6-F | AGATCAACATGCAAAGCACGTGA | Potri.001G042900 |
| HCT6-R | GCCAAAGTAACCAGGTGGGAGTTG |  |
| CSE1-F | CATCCAACCCTAGGAGATACAC | Potri.001G175000 |
| CSE1-R | TTCAAGCTCTTGTCCTCACTAG |  |
| CSE2-F | AACCATCCAAGCCCTACGATAA | Potri.003G059200 |
| CSE2-R | TTTGACCTTTTTATCCAACGGG |  |
| CCoAOMT1-F | CAGTAATTCAGAAAGCTGGTGTTGC | Potri.009G099800 |
| CCoAOMT1-R | GCATCCACAAAGATGAAATCAAAAC |  |
| CCoAOMT2-F | CCTTCCAACGCCAGGAAAGAGAGTA | Potri.001G304800 |
| CCoAOMT21-R | GTGGCCAACTTCTTGATGCCTTCCG |  |
| CCoAOMT3-F | TGAACAACTAGCACACGGAGT | Potri.008G136600 |
| CCoAOMT3-R | CGCCCTTCATCAACTGGCAC |  |
| CCR2-F | TCCCTGTCTGTTCTCCACTTTC | Potri.003G181400 |
| CCR2-R | ATCCAAGAAGCAATGAAACCAC |  |
| F5H1-F | GAGGTTGCTGGTTATTACGTTC | Potri.005G117500 |
| F5H1-R | CTGACCCAAACGGAATAAATTC |  |
| F5H2-F | TCGATGACATGCTAGCCTTTTA | Potri.007G016400 |
| F5H2-R | CTCTGGACTCTTCATTAGCTCC |  |
| COMT2-F | GCTTGTCCAGAACTGCTTATGA | Potri.012G006400 |
| COMT2-R | CACTCCACAAGTATCACCTTGC |  |
| CAD1-F | GATGTCTACACTGATGGCAAAC | Potri.009G095800 |
| CAD1-R | GAAACATGAACGAGGAACAGTA |  |
| CAD2-F | ACAGTATCAAGAATGACTGGGG | Potri.016G078300 |
| CAD2-R | GGAATCGAATGATGTAGCGTTC |  |
| CesA4-F | GCTTCAAGATGCATTGTAGAGG | Potri.002G257900 |
| CesA4-R | AATCTCAATAGAGCCTAGTGCC |  |
| CesA7A-F | AAACCAAGGGACCTGACACC | Potri.006G181900 |
| CesA7A-R | GGTGAACGCGTGATAAGCAA |  |
| CesA7B-F | CAAGCTTTGTATGGCTATGACC | Potri.018G103900 |
| CesA7B-R | CAACTGCACCATTCTTAGCATT |  |
| CesA8A-F | TCTGGGGCTAAACCTTCGGA | Potri.011G069600 |
| CesA8A-R | CAATGCAGAGGTGATGGCACA |  |
| CesA8B-F | GAAAACTTGTTGTTTGTGACGC | Potri.004G059600 |
| CesA8B-R | CAGAAGTGAACAAAGCCATTCA |  |
| IRX9-1-F | TGATCACAGTTTGAAGGGTTTAGC | Potri.006G131000 |
| IRX9-1-R | AAATTGAAGCCTTGCCCCCT |  |
| IRX9-2-F | TTCAGCAAACGAGAAGAAAGTG | Potri.016G086400 |
| IRX9-2-R | GTCTCGTCTTCAAGAGCTACTT |  |
| ACT1F | AAACTGTAATGGTCCTCCCTCCG | Potri.001G309500 |
| ACT1R | GCATCATCACAATCACTCTCCGA |  |
