# Supplementary figures and images for: The Inducible Accumulation of Cell Wall-Bound p-Hydroxybenzoates Is Involved in the Regulation of Gravitropic Response of Poplar
Source: Front Plant Sci. 2021 Dec 14;12:755576. doi: 10.3389/fpls.2021.755576 (PMC8712735; doi:10.3389/fpls.2021.755576)

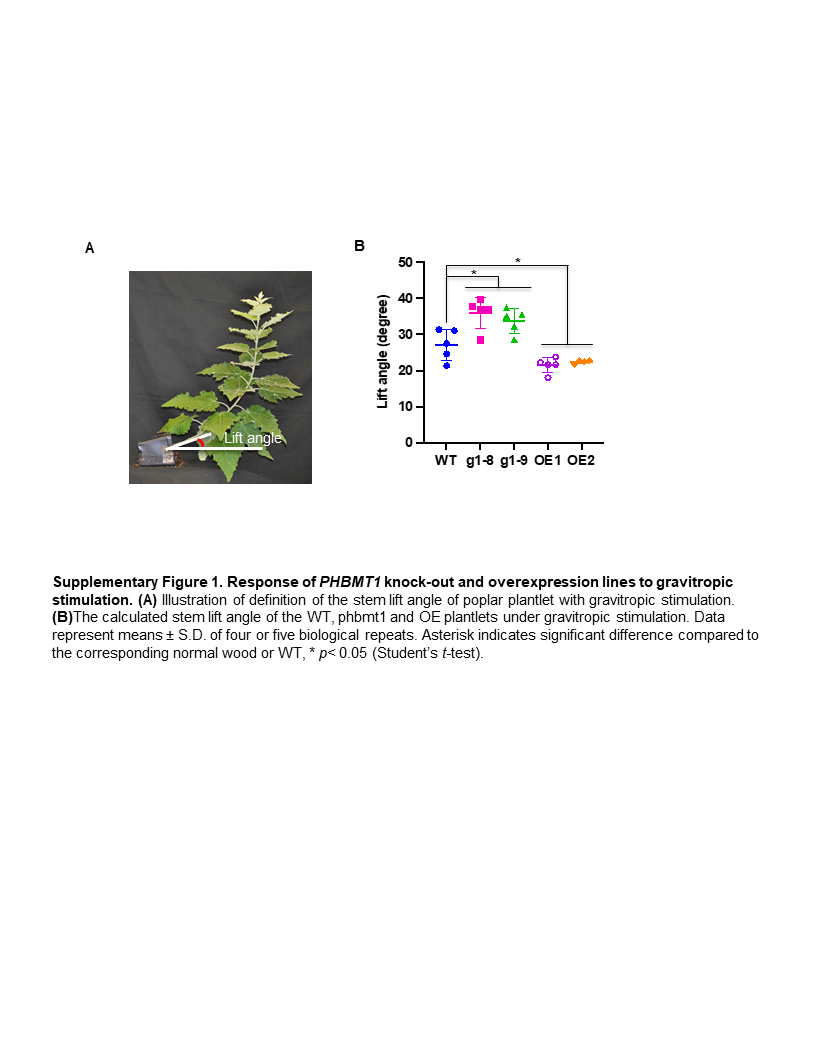

Supplement: Supplementary file 2 [file Image_1.tif]

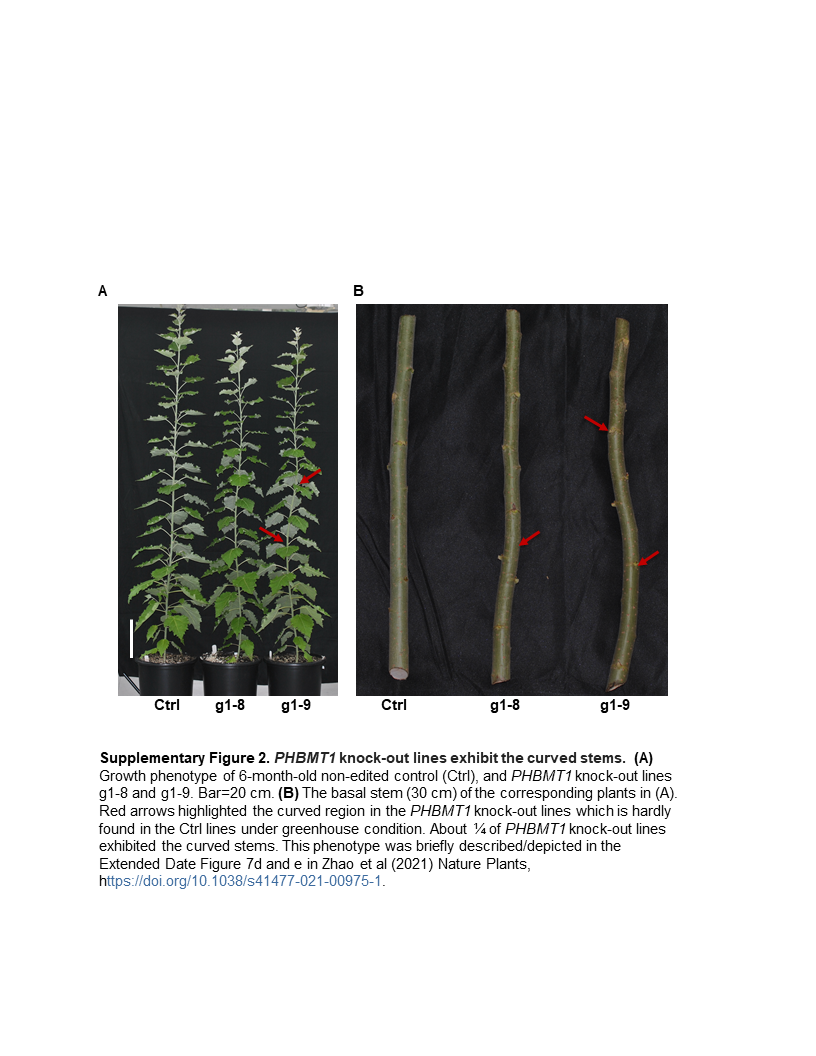

Supplement: Supplementary file 3 [file Image_2.tif]
